# Distinct physiological characteristics and altered glucagon signaling in GHRH knockout mice: Implications for longevity

**DOI:** 10.1111/acel.13985

**Published:** 2023-09-04

**Authors:** A. Tate Lasher, Liou Y. Sun

**Affiliations:** ^1^ Department of Biology University of Alabama at Birmingham Birmingham Alabama USA

**Keywords:** extended lifespan, glucagon, growth hormone, insulin sensitivity

## Abstract

Our previous research has demonstrated that mice lacking functional growth hormone‐releasing hormone (GHRH) exhibit distinct physiological characteristics, including an extended lifespan, a preference for lipid utilization during rest, mild hypoglycemia, and heightened insulin sensitivity. They also show a further increase in lifespan when subjected to caloric restriction. These findings suggest a unique response to fasting, which motivated our current study on the response to glucagon, a key hormone released from the pancreas during fasting that regulates glucose levels, energy expenditure, and metabolism. Our study investigated the effects of an acute glucagon challenge on female GHRH knockout mice and revealed that they exhibit reduced glucose production, likely due to suppressed gluconeogenesis. However, these mice showed an increase in energy expenditure. We also observed alterations in pancreatic islet architecture, with smaller islets and a reduction of insulin‐producing beta cells but no changes in glucagon‐producing alpha cells. Additionally, the analysis of hepatic glucagon signaling showed a decrease in glucagon receptor expression and phosphorylated CREB. In conclusion, our findings suggest that the unique metabolic phenotype observed in these long‐lived mice may be partly explained by changes in glucagon signaling. Further exploration of this pathway may lead to new insights into the regulation of longevity in mammals.

## Graphical abstract

Our past research showed that mice without functional growth hormone‐releasing hormone (GHRH) have distinct traits like a longer lifespan and lipid‐based energy use. Our new study on these mice's response to glucagon, a fasting‐related hormone, revealed reduced glucose production, increased energy expenditure, and changes in pancreatic architecture. These findings suggest altered glucagon signaling may contribute to their unique metabolism and extended lifespan.

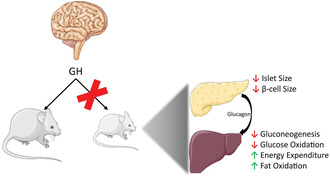

**Abbreviations**
ACACA: acetyl‐coenzyme A carboxylase alpha
ACACB: acetyl‐coenzyme A carboxylase beta
cAMP: cyclic adenosine monophosphate
CPT1a: carnitine palmitoyltransferase 1a
CPT2: carnitine palmitoyltransferase 2
CREB: cAMP response element binding protein
G6PC: glucose6phosphatase
GCG: glucagon
*Gcgr*
: glucagon receptor
GcgTT: glucagon tolerance test
GH—: growth hormone
GHR—: growth hormone receptor
GHRH—: growth hormonereleasing hormone
GHRHKO: GHRH knockout
GLUT: facilitated glucose transporter member
GlyTT: glycerol tolerance test
GTT: glucose tolerance test
INS: insulin
ITT: insulin tolerance test
PCK1: phosphoenolpyruvate carboxykinase 1
PCX: pyruvate carboxylase
PPARa: peroxisome proliferator activated receptor alpha
PTT: pyruvate tolerance test
RER: respiratory exchange ratio
VCO2: carbon dioxide production
VO2: oxygen consumption

## 1 INTRODUCTION

One of the most consistent means to extend the mammalian lifespan in laboratory settings is through genetic disruption of the murine growth hormone (GH) signaling pathway. Hypopituitary Ames dwarf and Snell dwarf mice, deficient in GH as well as other pituitary‐derived hormones, display markedly enhanced lifespans relative to their normal littermates (Brown‐Borg et al., 1996; Flurkey et al., 2001). Comparable extensions in longevity have been reported in mice lacking the GH receptor (GHR), GH‐releasing hormone (GHRH), and both the GHR and GHRH (Coschigano et al., 2000, 2003; Icyuz et al., 2021; Sun et al., 2013). The repeated observation of extended lifespan in the various GH‐disrupted mouse models across several laboratories and on multiple genetic backgrounds highlights the importance of this pathway in understanding endocrine regulators of mammalian longevity. In addition to extended lifespan, GH‐disrupted mice display a unique physiology that confers extended health span. These mice preferentially utilize lipids relative to carbohydrates during periods of rest/fasting, display mild hypoglycemia during fasting, are resistant to diabetes despite significantly elevated adiposity, and have a greater response to exogenous insulin (Icyuz et al., 2020; Liu et al., 2004; Longo et al., 2010; Sun et al., 2013; Westbrook et al., 2009). The heightened insulin sensitivity in these animals has been the subject of extensive study, however, the function of other pancreatic hormones has received considerably less attention in these models of healthy aging.

Glucagon is a 29‐amino acid peptide derived from the α‐cells within the pancreatic islets of Langerhans and is classically considered the counter to insulin, elevating gluconeogenesis and glycogenolysis during periods of fasting (Habegger et al., 2010). Fasting‐induced hypoglycemia and amino acid cues trigger the release of glucagon from the pancreas, where it enters systemic circulation (Ohneda et al., 1969; Unger et al., 1969). Canonical glucagon signaling involves glucagon binding the glucagon receptor, a G_S_ subtype of the G‐protein coupled protein receptor superfamily, triggering intracellular cAMP accumulation, and ultimately the phosphorylation of the cAMP response element binding protein (CREB) (Herzig et al., 2001; Jelinek et al., 1993). Phosphorylation of CREB at Ser 133 causes the expression of key gluconeogenic genes such as phosphoenolpyruvate carboxykinase 1, pyruvate carboxylase, and glucose‐6‐phosphatase (Altarejos & Montminy, 2011; Herzig et al., 2001; Koo et al., 2005), ultimately resulting in endogenous glucose production. Glucagon is also known to stimulate energy expenditure in both rodents (Davidson et al., 1960) and humans (Nair, 1987), and while the mediator for these effects appears to be hepatic in origin (Kim et al., 2018) a definitive mechanism for these observations remains elusive. More recently, glucagon has been shown to stimulate hepatic lipolysis and mitochondrial fatty acid oxidation in an inositol triphosphate receptor‐1‐dependent manner (Longuet et al., 2008; Zhang et al., 2020). The cumulative body of evidence indicates that glucagon is a crucial physiological regulator of metabolic function in mammals. Previous work from our group has shown that mice deficient in GH mediated by a deletion of the gene coding for GHRH (GHRH‐KO mice) display reductions in fasting glycemia, preference for lipid utilization relative to carbohydrates during periods of fasting, and significantly extended lifespan (Icyuz et al., 2020; Sun et al., 2013), Interestingly, calorie restriction further extends lifespan in these mice with a more dramatic effect in female mice (Sun et al., 2013). These findings suggest that there is a distinct response to fasting in these long‐lived animals.

In this study, we examined the response to glucagon in vivo, focusing our attention to the female sex for two reasons. First, the current body of literature mainly features male animals which introduces a sex‐specific bias in elucidating potential mechanisms for lifespan regulation. Secondly, the greater lifespan increase observed in calorie restricted female GHRH‐KO mice indicates the fasting response in these females is of relevance for understanding regulators of mammalian longevity. We show that in these GHRH‐KO females there is a reduced capacity for gluconeogenesis but greater sensitization to the metabolic rate‐increasing effect of glucagon. Based on these findings, it can be inferred that the distinctive metabolic characteristics observed in these animals are, to some extent, influenced by their specific glucagon signaling activity. Additionally, it is conceivable that the glucagon signaling pathway presents a new mechanism for regulating the aging process in mammals.

## 2 METHODS

### 2.1 Animals

Mice with a CRISPR/Cas9 mediated GHRH deletion have been previously described (Icyuz et al., 2020) and were maintained on a mixed BALB/cByJ X C57BL/6J genetic background to increase genetic diversity and increase fecundity. Mice were group housed at 5–7 individuals per cage in a pathogen‐free facility maintained on a standard 12‐h light and 12‐h dark cycle at 20–23°C. All animals had ad‐libitum access to standard rodent chow (NIH‐31 Rat and Mouse diet, 18% protein, 4% fat) and drinking water except where mice were fasted, as indicated in the results section. All mice used in this study were females between 5 and 7 months old at the time of experiments. All experimental protocols utilizing live animals were approved by the University of Alabama at Birmingham institutional animal care and use committee.

### 2.2 Substrate and hormone tolerance tests

For insulin tolerance tests (ITT) mice with ad‐libitum access to standard rodent diet were administered 1 IU/kg porcine insulin (Sigma‐Aldrich). Food was withdrawn immediately prior to ITT to prevent eating during the test. For glucose (GTT), pyruvate (PTT), and glycerol (GlyTT) tolerance tests overnight (16 h) fasted mice were administered 1 g/kg of the appropriate substrate. For glucagon tolerance tests (GcgTT) overnight fasted mice were administered 16 μg/kg glucagon (Sigma‐Aldrich). All substrates and hormones were administered by intraperitoneal injection. Tail prick blood glucose measurements were taken immediately before injection (“minute 0”) and at time points indicated following injection using an AgaMatrix PRESTO handheld blood glucometer.

### 2.3 Serum glucagon content analysis

Whole blood was collected from overnight (16–18 h) fasted mice via cardiac puncture after carbon dioxide‐induced deep anesthesia and cervical dislocation. Blood was allowed to clot at room temperature for 15 min then serum was separated by centrifugation for 10 min at 3000× ***g*** 4°C. Collected serum was stored at −80°C until analysis. Serum glucagon content was determined using a Mouse Glucagon ELISA kit (Crystal Chem, catalog# 81518) following the manufacturer protocol.

### 2.4 Immunofluorescent microscopy

Overnight (16–18 h) fasted animals were sacrificed by carbon dioxide‐induced deep anesthesia followed by cervical dislocation. Pancreas tissue was dissected and fixed in 4% paraformaldehyde for 24 h at 4°C. Fixed tissues were embedded in paraffin by the University of Alabama at Birmingham Comparative Pathology Laboratory. Six‐micrometer sections were deparaffinized, rehydrated, and blocked for 1 h at room temperature in 1% bovine serum albumin (BSA)/5% normal donkey serum in phosphate‐buffered saline (PBS). Sections were then incubated overnight at 4°C with antibodies against glucagon and insulin diluted in PBS containing 1% BSA. Slides were washed in PBS and then incubated for 2 h at room temperature with Alexa Fluor 488‐ and Cy3‐conjugated secondary antibodies. Islets were imaged using a Leica DMI8 fluorescent microscope with a motorized stage. Images were analyzed using NIH ImageJ software. Antibody information is provided in Table S1.

### 2.5 RNA extraction and RT‐qPCR

Overnight (16–18 h) fasted animals were sacrificed by carbon dioxide‐induced deep anesthesia followed by cervical dislocation. Liver tissue was dissected and total RNA was extracted using TRIzol (Invitrogen) following manufacturer protocol. Contaminating DNA was removed using RNase‐free DNase I (New England Biolabs) treatment following manufacturer protocol. A high‐capacity cDNA reverse transcription kit (Applied Biosystems) was used to reverse transcribe 1.3 μg DNase I‐treated RNA to cDNA following manufacturer protocol. Real‐time qPCR was carried out using Luna Universal qPCR Master Mix (New England Biolabs) and a QuantStudio 3 thermal cycler (Applied Biosystems). The fold change in gene expression was calculated using the 2^−ddCt^ calculation and was normalized to the wild‐type group. Beta‐actin (ACTB) was used as endogenous control. Primer sequences are provided in Table S2.

### 2.6 Western blotting

Liver tissue collected from overnight (16–18 h) fasted mice was homogenized in lysis buffer (20 mM Tris pH 6.8, 1% SDS, 10% Glycerol, 4 mM DTT) supplemented with Pierce protease (Thermo) and Pierce phosphatase inhibitor tablets (Thermo) then centrifuged for 10 min at 16,000 × ***g*** 4°C. The supernatant was collected, and protein concentration was determined by DC protein assay (BioRad). Twenty micrograms of total protein was added to Laemmli sample buffer containing 5% 2‐mercaptoethanol, heated to 95°C for 5 min, separated by SDS‐PAGE then electrotransferred to a 0.45 μm nitrocellulose membrane. Nonspecific binding was blocked with tris‐buffered saline containing 0.1% Tween‐20 (TBST) and 5% BSA for 1 h at room temperature. Membranes were probed with primary antibodies diluted in blocking buffer for 2 h at room temperature, washed briefly with TBST, then incubated with horseradish peroxidase‐conjugated secondary antibodies diluted in blocking buffer for 1 h at room temperature. Protein signals were developed using SuperSignal West Pico Plus chemiluminescent substrate (Thermo) and imaged using a SynGene PXi imager. Protein band density was quantified using NIH ImageJ software. Antibody information is provided in Table S1. Uncropped membranes are provided in supplement.

### 2.7 Indirect calorimetry

All experiments utilizing indirect calorimetry employed the comprehensive lab animal monitoring system (CLAMS; Columbus Instruments), which collects oxygen consumption (VO_2_) and carbon dioxide production (VCO_2_) data every 9 min continuously for each mouse housed. The respiratory exchange ratio (RER) and energy expenditure were calculated as previously described (Icyuz et al., 2020). The rate of glucose oxidation was calculated as 4.57(VCO_2_)–3.23(VO_2_) and the rate of fat oxidation was calculated as 1.69(VO_2_)–1.69(VCO_2_) as published by Franczyk and colleagues (Franczyk et al., 2021), where VO_2_ and VCO_2_ are expressed as ml/kg/h. For experiments investigating the effect of acute glucagon treatment, mice were fasted overnight (16–18 h) in their home cages and then individually placed into indirect calorimetry chambers. Mice were acclimated for approximately 30 min where no data was collected and then injected intraperitoneally with 16 μg/kg glucagon or an equivalent volume of PBS between the first (“minute −9”) and second (“minute 0”) recording. Data were recorded for a total of 90 min for these experiments. For longer‐term indirect calorimetry experiments, mice were individually housed in indirect calorimetry chambers with ad‐libitum access to standard rodent diet and water for an acclimation period of 3 days where no data were collected. Data were collected every 9 min for each animal over the next 3 days, with data being averaged into hourly values for presentation.

### 2.8 Statistical analysis

Two group means were compared using the unpaired two‐tailed *t*‐test with the welch correction applied. Where several means were compared, factorial repeated measure ANOVA was used, with post hoc testing carried out where significant effects were detected. The Benjamini and Hochberg correction or Tukey correction was applied to post hoc tests as indicated. Means were considered significantly different at *p* < 0.05. Analyses were carried out and figures were generated using the R programming language.

## 3 RESULTS

**FIGURE 1:**
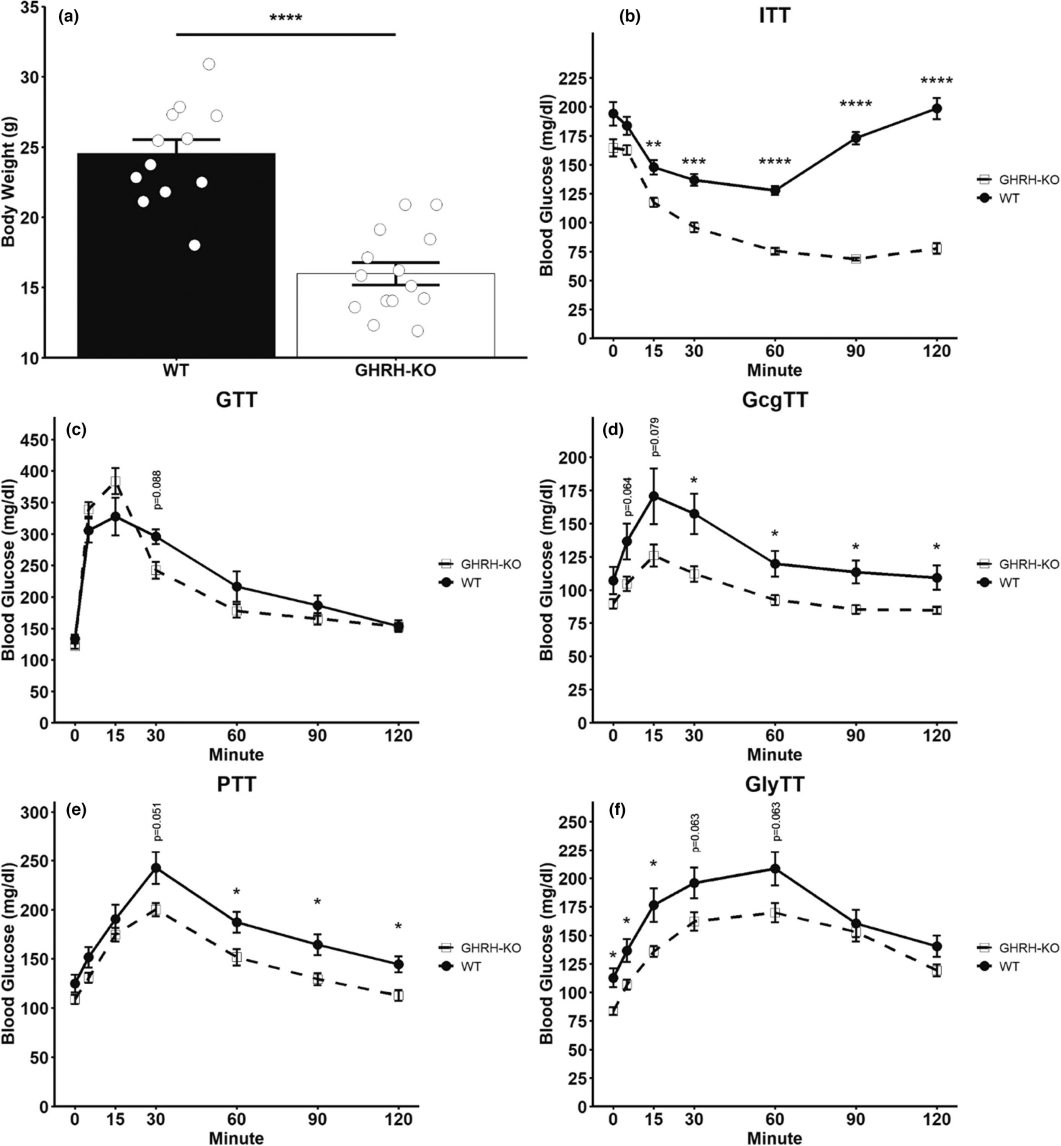
Differential capacity for endogenous glucose production in female GHRH‐KO mice. Body weight in female GHRH‐KO and WT littermates (a). 1 IU/kg insulin (b), 1 g/kg glucose (c), 16 μg/kg glucagon (d), 1 g/kg pyruvate (e), and 1 g/kg glycerol (f) tolerance tests in GHRH‐KO and WT females. Intraperitoneal injection was used to administer all agents. Animals were overnight fasted (approx. 16 h) except in (c) where animals had ad‐lib access to standard diet until the start of testing. Data presented as mean ± SEM with individual data points representing individual mice. *p < 0.05; **p < 0.01; ***p < 0.001; ****p < 0.0001. Statistical significance was determined by two‐tailed Student's *t*‐test with the welch correction applied (a) or by factorial repeated measure ANOVA followed by Benjamini–Hochberg adjustment for multiple comparisons. *N* = 5‐7(b, c) or *N* = 12–14 (a, d–f).

Expectedly, our GHRH‐KO animals had significantly reduced bodyweight compared to their WT littermates (Figure 1A), along with significantly reduced blood glucose following an insulin challenge at all time points assessed from 15 min post insulin challenge and beyond (Figure 1B), consistent with our previous report of enhanced insulin sensitivity in these mice (Icyuz et al., 2020). We also observed marginal differences to blood glucose excursion following a glucose challenge with a trend toward reduced glycemia in GHRH‐KO mice observed at 30 min post glucose challenge (*p* = 0.066) but no differences observed at any other time points assessed (Figure 1C), consistent with our previous work (Icyuz et al., 2020; Sun et al., 2013). To directly assess the glycemic response to the fasting hormone glucagon, we carried out a glucagon tolerance test (GcgTT) in overnight fasted mice, where administration of 10–20 μg/kg glucagon has been consistently reported to elevate glycemia (Capozzi et al., 2019; Chen et al., 2005; Gelling et al., 2003; Rossi et al., 2018). We observed a significantly reduced glycemic response to glucagon in GHRH‐KO mice from 30 min post glucagon challenge and beyond, with trends toward a reduced response observed at 5 (*p* = 0.064) and 15 (*p* = 0.079) min post glucagon challenge as well (Figure 1D). As glucagon is a potent regulator of gluconeogenesis, we carried out pyruvate tolerance tests (PTT) and glycerol tolerance tests (GlyTT) to gauge gluconeogenesis. PTT revealed a significantly lower glycemic response at 60, 90, and 120 min following a pyruvate challenge, with a trend for a reduction in this response at 30 min (*p* = 0.051) observed as well (Figure 1E). GlyTT also revealed a dampened glycemic response until 15‐min following glycerol administration, with a trend for reduction in this response at the 30‐ and 60‐min time points (Figure 1F). Taken together, these data demonstrate that female GHRH‐KO mice display impaired endogenous glucose production in response to glucagon resulting from reduced gluconeogenic activity.

**FIGURE 2:**
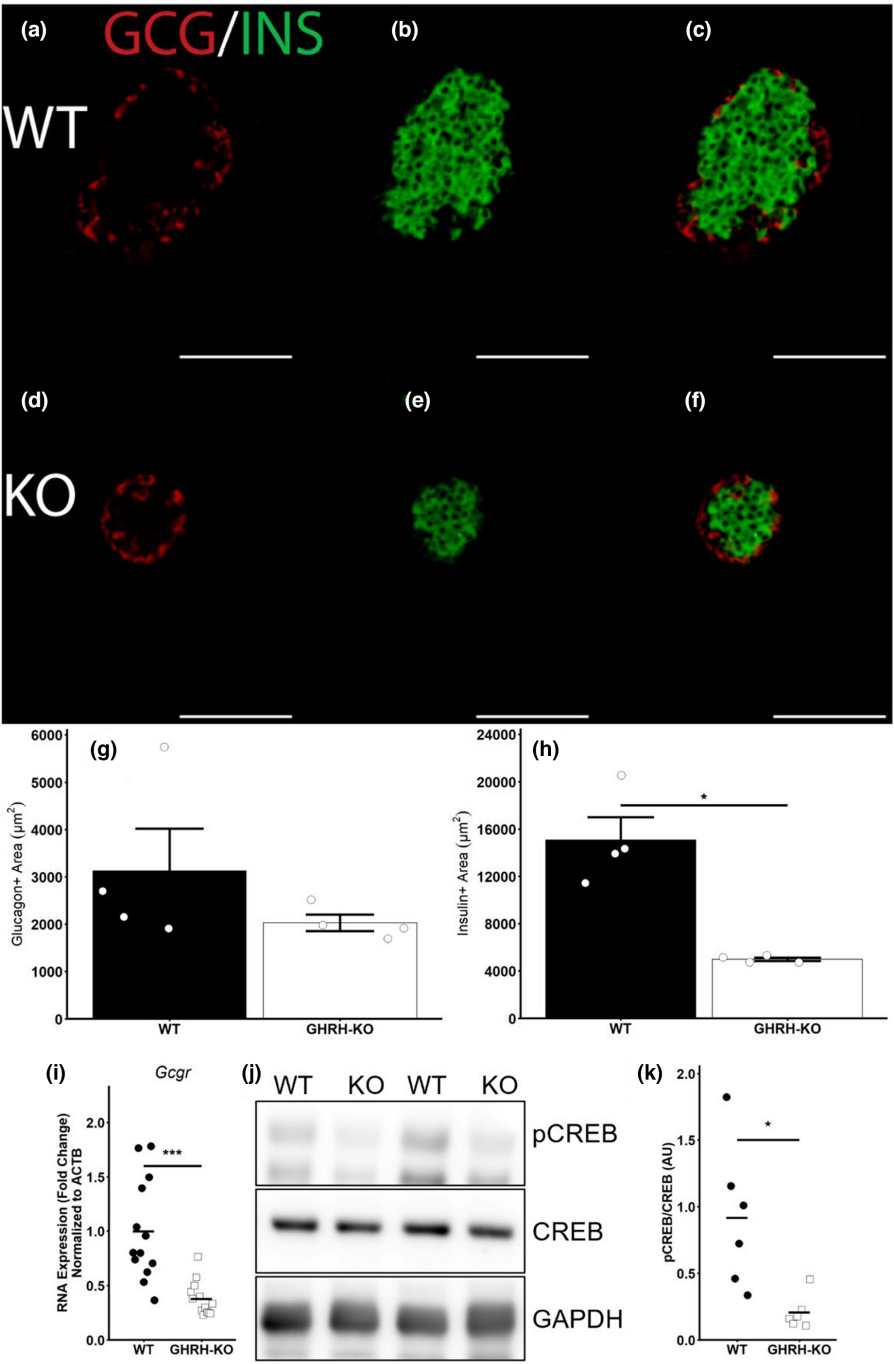
Altered islet architecture in GHRH‐KO females. Representative images of glucagon (a), insulin (b), and merged (c) immunofluorescent staining in wild‐type pancreatic islets. Representative images of glucagon (d), insulin (e), and merged (f) immunofluorescent staining in GHRH‐KO pancreatic islets. Quantification of glucagon‐staining area (g), insulin staining area (h), hepatic mRNA expression of the glucagon receptor (*Gcgr*; i), and representative western blots of phosphorylated CREB, total CREB, and GAPDH in the liver of overnight (16–18 h) fasted GHRH‐KO and WT females (j). Quantification of the phosphorylated CREB to total CREB ratio is presented (k). Scale bars represent 100 μm. Data presented as mean ± SEM, or as group means with individual points representing individual mice. Individual data points in G‐H represent the average of all islets quantified for an individual. 6–10 islets were quantified per mouse per genotype. *p < 0.05; ***p < 0.001. Statistical significance was determined by two‐tailed Student's *t*‐test with the welch correction applied. *N* = 4 mice per group (g, h), *N* = 13 mice per group (i), or *N* = 6 mice per group (k).

As glucagon is derived from pancreatic islets of Langerhans, we carried out immunofluorescent microscopy to assess pancreatic islet morphology in our GHRH‐KO mice. While GHRH‐KO islets had a lower mean glucagon‐staining area, this difference was not significant (Figure 2A,D,G) and no difference in serum glucagon levels were detected (Figure S1A). The insulin‐staining area, however, was dramatically reduced in GHRH‐KO mice (Figure 2B,E,H), consistent with our previous report of reduced plasma insulin content in GHRH‐KO mice (Icyuz et al., 2020). We also detected significantly reduced pancreatic islet area in GHRH‐KO mice (Figure S1B) which is consistent with our group's previous report (Icyuz et al., 2020). Also noteworthy is the significant reduction in the ratio of glucagon‐stained area to insulin‐stained area in GHRH‐KO mice (Figure S1C). We also detected significantly reduced glucagon receptor (*Gcgr*) mRNA expression in fasted GHRH‐KO liver tissue (Figure 2I). To investigate the canonical glucagon receptor signaling pathway more completely in these animals we assessed the phosphorylation of CREB, a key mediator of glucagon‐induced transcription of gluconeogenic genes, by western blot analysis. Fasted GHRH‐KO livers displayed a significantly lower ratio of phosphorylated CREB/total CREB (Figure 2J,K). In summary, our microscopy data show that GHRH‐KO mice possess smaller pancreatic islets resulting from fewer insulin‐producing beta cells without significant loss of glucagon‐producing alpha cells. Gene expression and western blot analysis indicate a reduced capacity for responding to glucagon, as indicated by lower *Gcgr* expression and reduced CREB phosphorylation.

**FIGURE 3:**
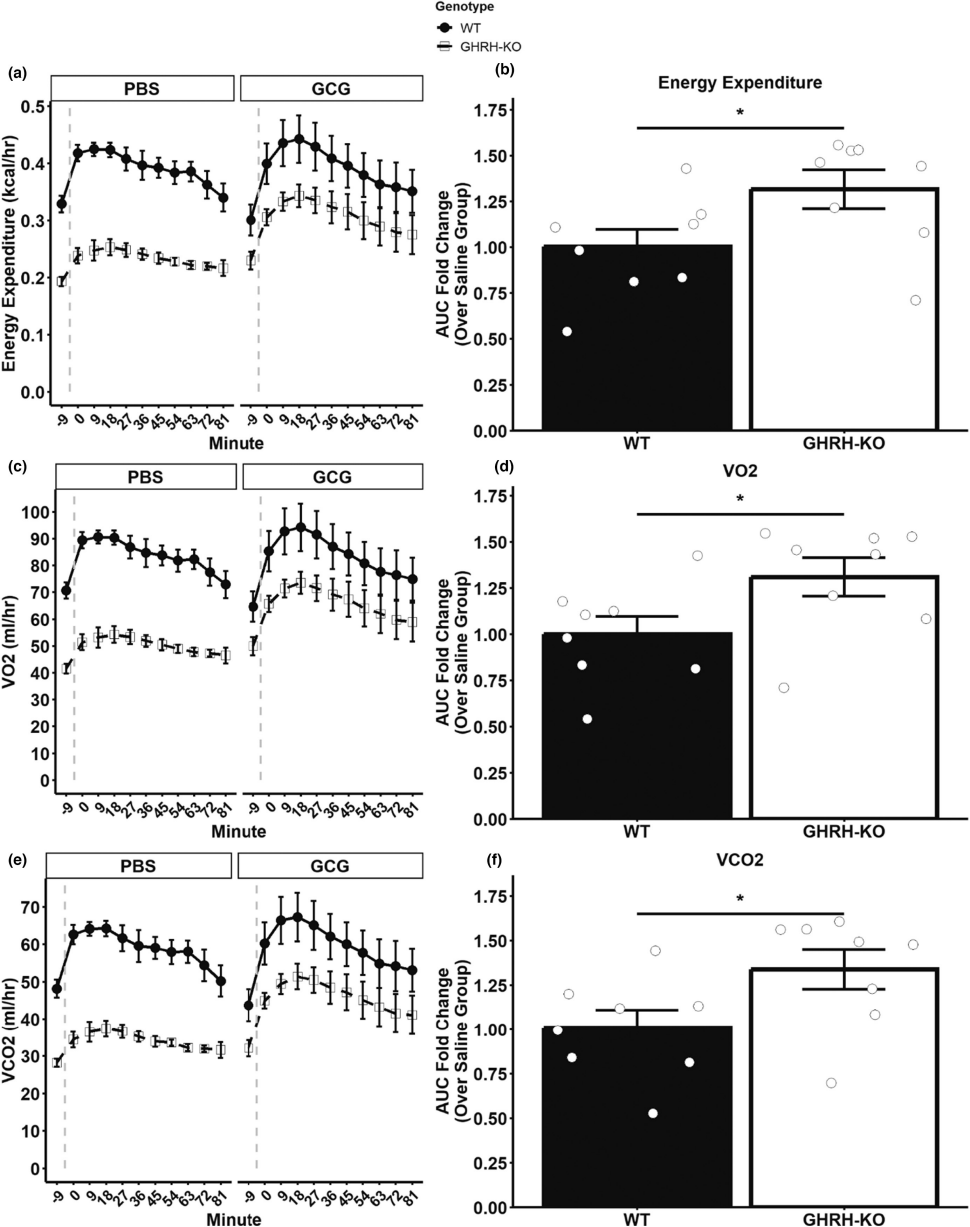
Elevated glucagon‐induced energy expenditure in GHRH‐KO females. Overnight fasted (16–18 h) animals were administered 16 μg/kg glucagon (GCG) or an equivalent volume of phosphate buffered saline (PBS) by intraperitoneal injection. Vertical dashed lines indicate injection time. Energy expenditure (a), oxygen consumption (VO_2_) (C), and carbon dioxide production (VCO_2_) (e) were measured by indirect calorimetry. Area under the curve (AUC) was calculated for each parameter, and comparisons were made by normalizing the glucagon treated group to the PBS treated group for both genotypes (B, D, F) in order to account for differences in metabolic rate due to body size. Data presented as mean ± SEM with individual data points representing individual mice. *p < 0.05. Statistical analysis was carried out by two‐tailed Student's *t*‐test with the welch correction applied. *N* = 6 (PBS) or 8 (glucagon).

Glucagon has long been known to stimulate energy expenditure in laboratory rodents (Davidson et al., 1960). To gauge the metabolic response of our GHRH‐KO mice to glucagon, we administered an acute 16 μg/kg glucagon challenge, mimicking our glucagon tolerance test protocol, or an equivalent volume of phosphate‐buffered saline (PBS) to overnight fasted mice in the context of indirect calorimetry. Shockingly, glucagon treatment stimulated a greater increase in energy expenditure of GHRH‐KO mice compared to WT littermates (Figure 3A). To quantify this observation, we calculated the area under the curve (AUC) for each glucagon‐treated group and normalized this to the AUC of the genotype‐matched PBS‐treated group. The glucagon‐treated WT animals displayed an approximately onefold change in the energy expenditure AUC compared to the PBS‐treated WT animals, indicating no appreciable change in metabolic rate at this dosage. The glucagon‐treated GHRH‐KO animals, on the other hand, demonstrated an approximately 1.3‐fold change in this parameter which was significantly greater compared to the WT animals (Figure 3B). Similar patterns were observed in the VO_2_ and VCO_2_ parameters, with glucagon treatment stimulating significant increases in these respiratory measurements in GHRH‐KO, but not WT animals (Figure 3C–F). These data show GHRH‐KO females are hypersensitive to the metabolic rate elevating effects of exogenous glucagon.

**FIGURE 4:**
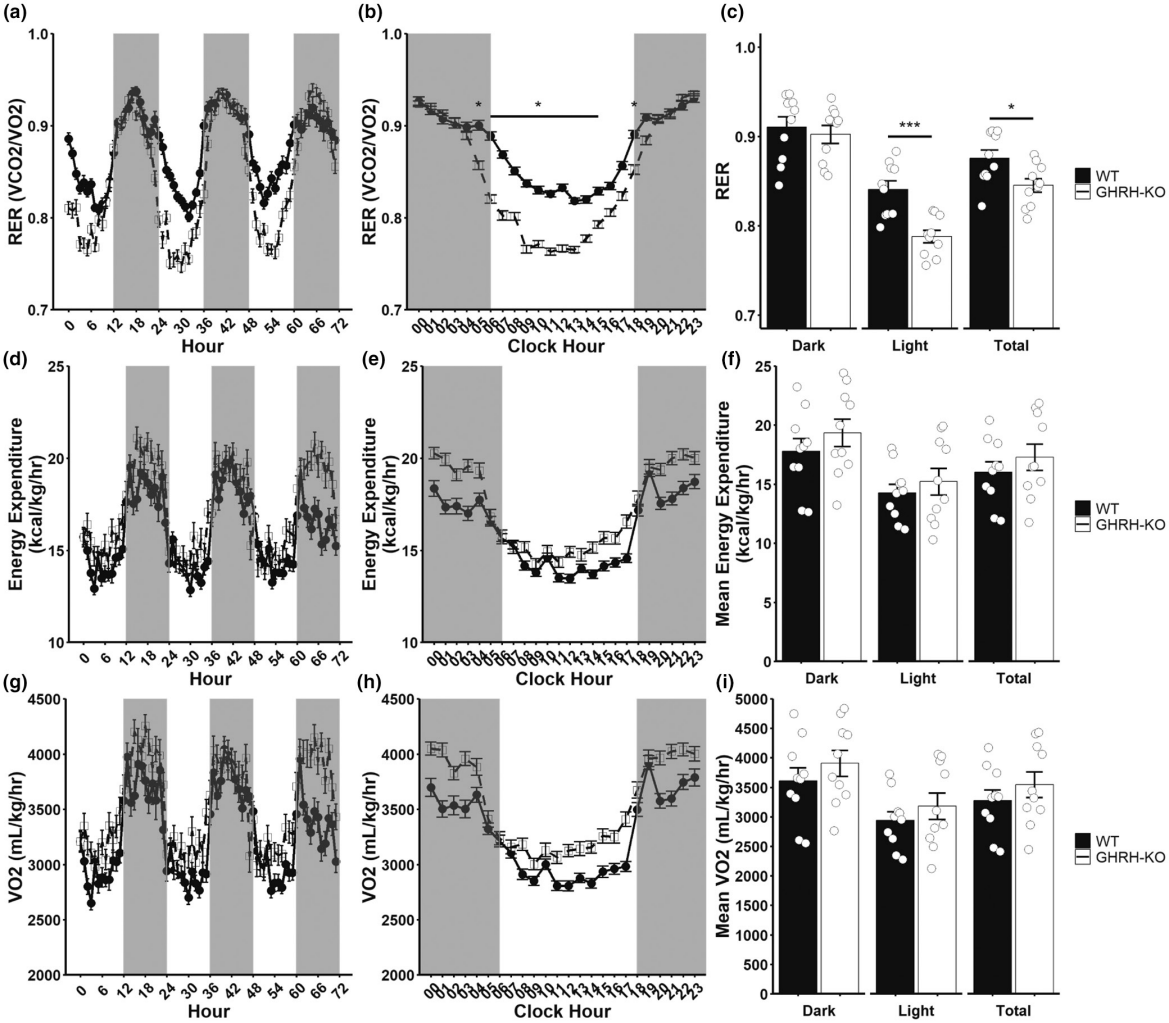
Reduced respiratory exchange ratio (RER) in GHRH‐KO females. Indirect calorimetry data for female GHRH‐KO and WT mice with ad‐lib access to standard diet. RER (a), body weight normalized energy expenditure (D), and body weight normalized oxygen consumption (VO_2_; g) for the entire 3‐day data collection period. The average RER (b), body weight normalized energy expenditure (e), and body weight normalized VO_2_ (h) values for each hour in a single day. Comparison of mean RER (c), body weight normalized energy expenditure (f), and body weight normalized VO_2_ (i) for the 3‐day data collection period. Data presented as mean ± SEM with individual data points representing individual mice. *p < 0.05; ***p < 0.001. Statistical analysis was carried out by factorial repeated measure ANOVA followed by Tukey adjustment for multiple comparisons (b, e, h) or by two‐tailed Student's *t*‐test with the welch correction applied (c, f, i). *N* = 10 per group.

We also employed longer‐term indirect calorimetry to assess the metabolic disposition of these animals under conditions of minimal experimenter manipulation. Consistent with our previous reports (Icyuz et al., 2020; Sun et al., 2013), GHRH‐KO mice display reduced respiratory exchange ratio (RER) (Figure 4A). Pairwise comparisons of hourly RER values revealed significantly reduced RER, primarily during light hours (Figure 4B). Comparisons of mean RER values during the dark cycle, light cycle, and total duration of indirect calorimetry revealed significantly reduced mean RER during the total and light cycles (Figure 4C), indicating elevated utilization of lipids relative to carbohydrates during the light cycle. Despite these changes in RER, no appreciable differences in body weight normalized energy expenditure (Figure 4D–F) or in body weight normalized VO_2_ (Figure 4G–I), again consistent with previous reports from our lab (Sun et al., 2013). When data were analyzed by ANCOVA with body weight as a covariate, we detected significant reductions in energy expenditure and VO_2_ (Figure S2), consistent with our previous report (Icyuz et al., 2020). These data indicate that the most notable differences in the whole‐body metabolism occur during the post‐prandial light cycle, with a preference for lipids and reduced utilization of carbohydrates in GHRH‐KO mice occurring during this time.

**FIGURE 5:**
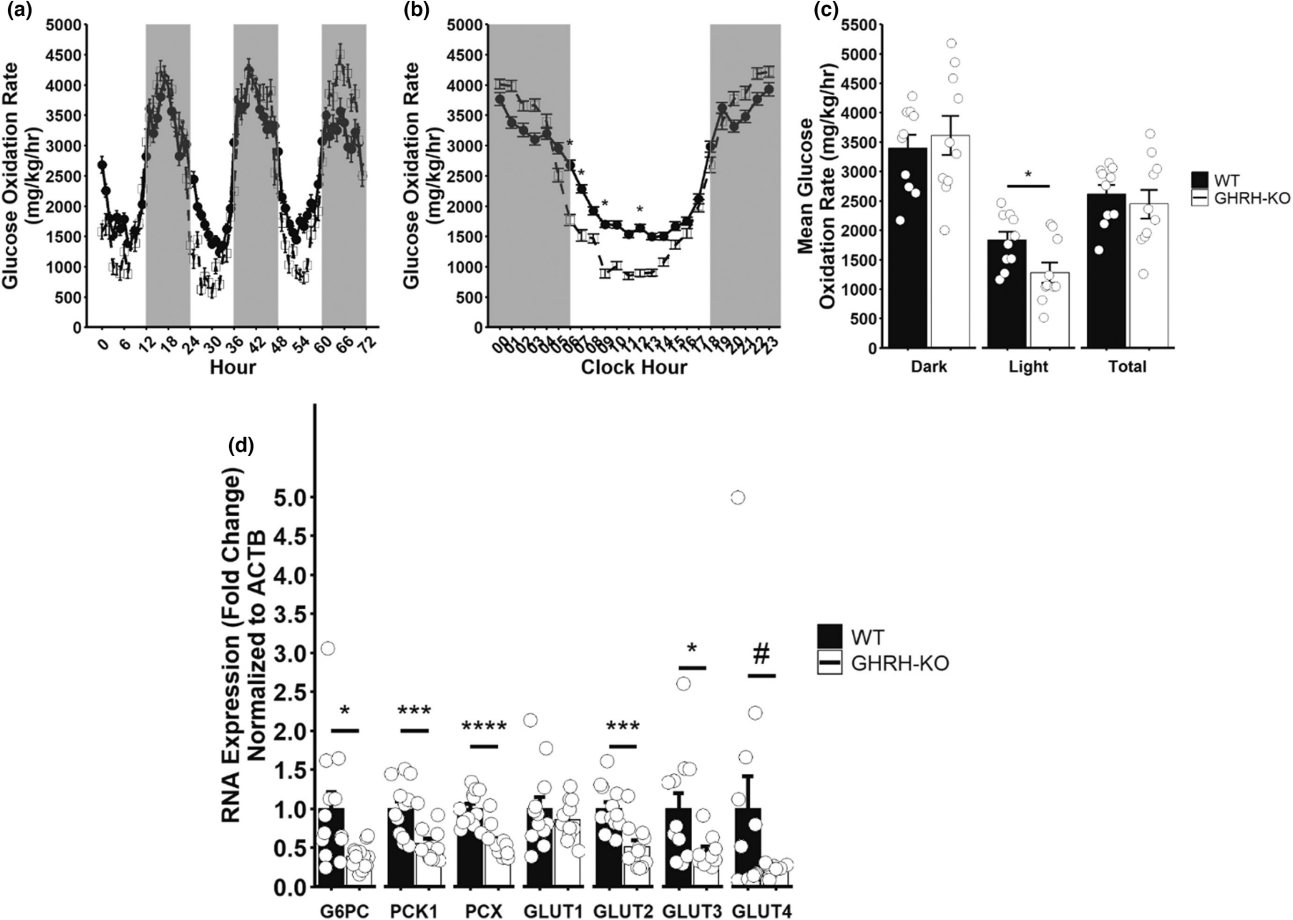
Reduced glucose oxidation and hepatic glucose production in GHRH‐KO females. Bodyweight normalized glucose oxidation rate for the entire 3‐day data collection period (a), average bodyweight normalized glucose oxidation rate for each hour in a single day (b), and mean bodyweight normalized glucose oxidation rate for the 3‐day data collection period (c). mRNA expression of genes related to hepatic glucose production and glucose transport in livers of overnight (16–18 h) fasted mice (d). Data presented as mean ± SEM with individual data points representing individual mice. #*p* < 0.1; *p < 0.05; **p < 0.01; ****p < 0.0001. Statistical analysis was carried out by factorial repeated measure ANOVA followed by Tukey adjustment for multiple comparisons (b) or by two‐tailed Student's *t*‐test with the Welch correction applied (c, d). *N* = 9–13 per group. G6PC glucose‐6‐phosphatase; PCK1 phosphoenolpyruvate carboxykinase 1; PCX pyruvate carboxylase; GLUT1‐4 facilitated glucose transporter member 1–4.

To investigate the contribution of glucose utilization to the reduced RER we observed during our indirect calorimetry experiment, we quantified the glucose oxidation rate according to previously published protocols (Franczyk et al., 2021). GHRH‐KO mice displayed reduced bodyweight normalized glucose oxidation rates (Figure 5A), with pairwise comparisons revealing significantly reduced body weight normalized glucose oxidation rates at hours 6, 7, 9, and 12 in GHRH‐KO mice (Figure 5B). Further, mean body weight normalized glucose oxidation rate was significantly reduced during the light cycle for GHRH‐KO animals (Figure 5C). When no adjustment for bodyweight was made, we observed dramatically reduced glucose oxidation rates in GHRH‐KO mice, and taking bodyweight into account by ANCOVA analysis revealed a significant effect of genotype (Figure S3A,B). To find a mechanistic explanation for this observation, we carried out RT‐qPCR on livers from overnight fasted animals and found that mRNA transcripts for the key gluconeogenic genes glucose‐6‐phosphatase, phosphoenolpyruvate carboxykinase‐1, and pyruvate carboxylase were significantly down‐regulated in GHRH‐KO mice (Figure 5D). Additionally, we also detected significant reductions in mRNA transcripts of the glucose transporters GLUT2 and GLUT3, as well as a trend (*p* = 0.092) for reduced GLUT4 expression (Figure 5D). These data indicate that the reduction in gluconeogenesis observed in GHRH‐KO mice is relevant under physiological conditions and that these mice display a lower capacity to transcribe key gluconeogenic genes during fasts.

**FIGURE 6:**
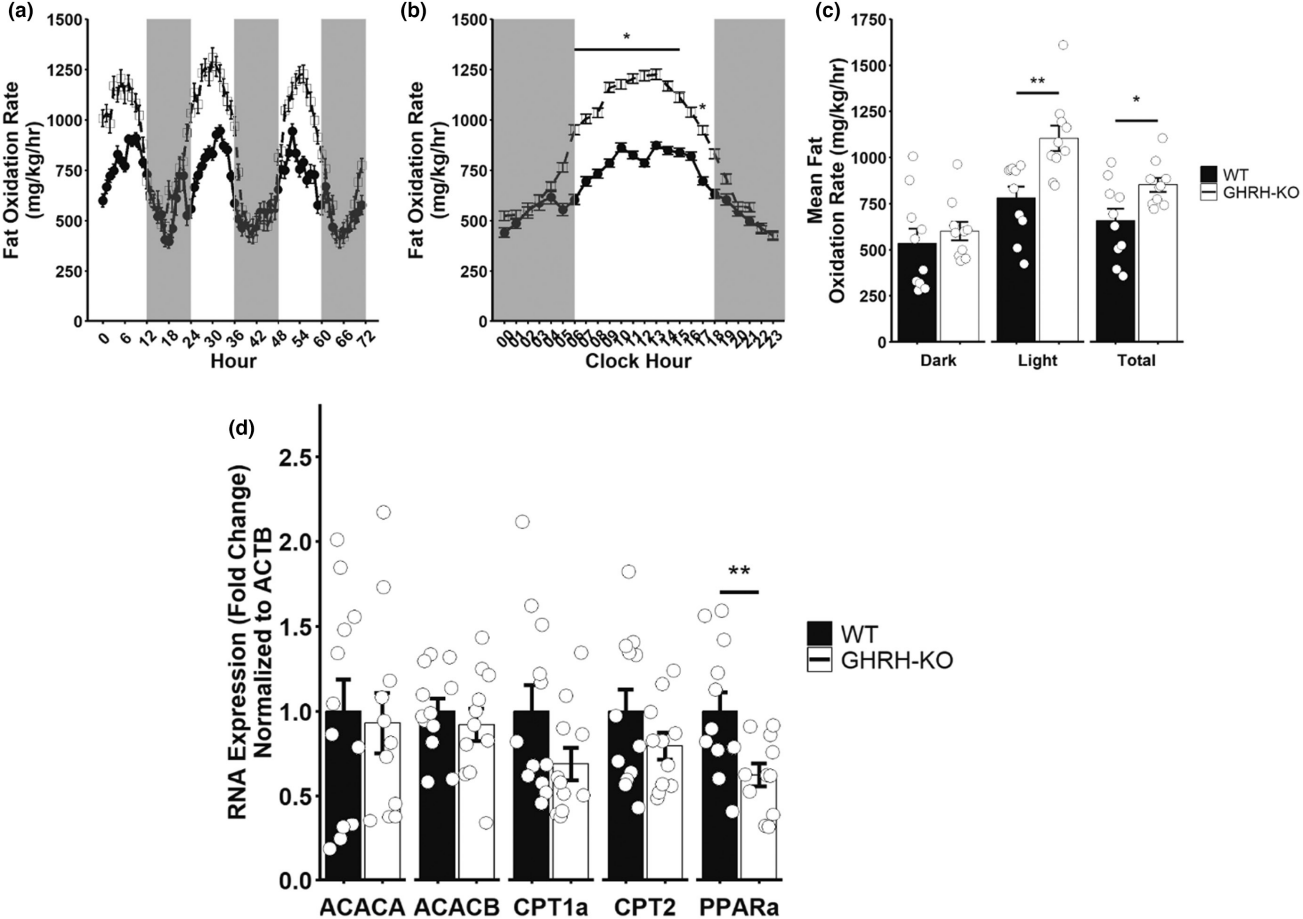
Elevated fat oxidation in GHRH‐KO females. Bodyweight normalized fat oxidation rate for the entire 3‐day data collection period (a), average bodyweight normalized fat oxidation rate each hour in a single day (b), and mean bodyweight normalized fat oxidation rate for the 3‐day data collection period (c). mRNA expression of genes related to hepatic beta‐oxidation in livers of overnight (16–18 h) fasted mice (d). Data presented as mean ± SEM with individual data points representing individual mice. *p < 0.05; **p < 0.01. Statistical analysis was carried out by factorial repeated measures ANOVA followed by Tukey adjustment for multiple comparisons (b) or by two‐tailed Student's *t*‐test with the welch correction applied (c, d). *N* = 9–13 per group. ACACA acetyl‐coenzyme A carboxylase alpha; ACACB acetyl‐coenzyme A carboxylase beta; CPT1a‐2 carnitine palmitoyltransferase 1a‐2; PPARa peroxisome proliferator activated receptor alpha.

We also quantified the fat oxidation rate as previously reported (Franczyk et al., 2021) to investigate the contribution of fat oxidation to the reduced RER we observed. GHRH‐KO females display significantly elevated bodyweight normalized fat oxidation rates during light hours compared to WT littermates, but comparable rates during dark hours (Figure 6A). Pairwise comparisons revealed significantly elevated fat oxidation at hours 6–15 and hour 17 in GHRH‐KO mice (Figure 6B). Mean bodyweight normalized fat oxidation was significantly greater during the duration of the experiment due to the dramatically elevated mean light cycle bodyweight normalized fat oxidation rate (Figure 6C). When no adjustment for bodyweight was made, we observed no discernable differences in fat oxidation rates between GHRH‐KO and WT mice, and ANCOVA analysis confirmed this observation as no effect of genotype was detected (Figure S3C,D). To investigate a mechanism for these changes, we carried out RT‐qPCR to quantify mRNA levels for genes associated with glucagon‐induced hepatic lipid metabolism (Longuet et al., 2008; Perry et al., 2020). Peroxisome proliferator‐activated receptor alpha expression was reduced in fasted GHRH‐KO female livers, however, we did not detect changes in mRNA transcripts coding for the key beta‐oxidation enzymes acetyl‐Coenzyme A carboxylase alpha/beta or carnitine palmitoyltransferase 1a/2 (Figure 6D). Taken together, these data indicate that GHRH‐KO females have elevated rates of fat oxidation in vivo, but that differences in hepatic transcription of beta‐oxidation genes do not account for these changes.

## 4 DISCUSSION

Identification of the unique physiological response to endocrine signals in long‐lived model organisms is critical for a mechanistic understanding of lifespan regulation. Here, we show that long‐lived GHRH‐deficient female mice display a reduced glycemic response to glucagon and impaired capacity for gluconeogenesis as indicated by reduced glycemic responses to pyruvate or glycerol challenges. Lower expression of key gluconeogenesis genes was also detected in fasted GHRH‐KO mice, indicating these changes were not artifacts of exogenous hormone administration. Consistent with this, we show that the canonical hepatic glucagon receptor signaling pathway is dampened in these mice as evidenced by lower glucagon receptor expression and lower CREB phosphorylation despite comparable alpha‐cell area. These findings were also observed under standard conditions, evidenced by the reduced RER and glucose oxidation during the light cycle, a typical fasting period for rodents. This indicates that this differential response to glucagon is physiologically relevant as it was seen across varying degrees of experimenter manipulation. Despite insensitivity to the glycemia‐elevating effect of glucagon, GHRH‐KO females displayed hypersensitivity to the metabolic‐rate elevating effects of glucagon. Also noteworthy was the significantly elevated fat oxidation in GHRH‐KO mice, however, these effects do not appear to be mediated by differential glucagon signaling, at least at the transcriptional level.

Lower utilization of glucose has been reported in several mouse models with extended longevity. When the respiratory exchange ratio is employed as a gauge of glucose utilization, hypopituitary Ames dwarf and GHR‐KO mice display lower glucose utilization compared to control animals. In the same study transgenic bGH mice, a model of GH overexpression also displaying reduced lifespan, display elevated respiratory exchange ratios when fasted (Westbrook et al., 2009), suggesting that the action of GH directly influences whole‐body glucose utilization. Our group has previously shown that mice deficient in GHRH display similar reductions in respiratory exchange ratios during the light cycle, (Icyuz et al., 2020; Sun et al., 2013) typically a period of fasting for laboratory rodents. The commonality of this observation in several long‐lived models suggests that reduced glucose utilization is a key feature of metabolism in these healthy‐aging mammals. Our data offers a novel mechanistic insight into the aforementioned observations. These GHRH‐KO mice exhibited a resistance to glucagon‐stimulated glucose production, along with decreased transcription of gluconeogenesis genes. As a result, they had to rely on alternative substrates, such as lipids, to meet their energy requirements. Impaired glucagon signaling, achieved through genetic knockout or pharmacological inhibition, has consistently been shown to increase insulin sensitivity. Glucagon receptor knockout mice display reduced glycemia in response to exogenous insulin (Gelling et al., 2003) and require greater amounts of glucose to maintain euglycemia during hyperinsulinemic‐euglycemic clamp experiments (Longuet et al., 2013; Sørensen et al., 2006). These improvements in peripheral insulin sensitivity have also been reported in mice where glucagon receptor signaling has been pharmacologically inhibited (McShane et al., 2016; Sharma et al., 2018). When liver‐specific glucagon receptor knockout models are employed, insulin sensitivity is increased to a level comparable to global knockouts (Longuet et al., 2013), indicating that hepatic glucagon receptor signaling is the primary mediator for these benefits. The impaired glucagon signaling seen in GHRH‐KO mice may be a contributing factor to the improved insulin sensitivity we have previously reported in this model (Icyuz et al., 2020), and potentially other GH‐disrupted models as well.

While we found that our GHRH‐KO animals were resistant to the glycemia‐elevating effects of glucagon, we also show that they display hypersensitivity to the metabolic‐rate elevating effects of glucagon. In our study, we detected elevated energy expenditure with an acute dose of 16 μg/kg, considerably less than the dosages of 0.5–1 mg/kg employed by others (Beaudry et al., 2019; Davidson et al., 1960). We hypothesize that this may be a feature of the heightened metabolic flexibility seen in GH‐attenuated mice, which is thought to contribute to their health span (Hong et al., 2014). At present, this hypothesis is difficult to test as a mechanism for glucagon‐induced metabolic rate elevation remains elusive and maybe the net sum of many small elevations in various catabolic processes (Kleinert et al., 2019), with assessment of a single process being insignificant on its own. It is also possible that extrahepatic glucagon receptor activity may have contributed to this observation. Of particular interest would be the contribution of glucagon receptor signaling in adipose tissue in this model. While the glucagon receptor has been detected in brown adipose, it does not appear to be necessary for glucagon's regulation of metabolic rate (Beaudry et al., 2019). In GH‐deficient mice however, brown adipose tissue is considerably more metabolically active as mRNA transcripts for key thermogenic and lipolytic genes are significantly elevated, and brown adipose tissue removal normalizes many metabolic features in these mice (Darcy et al., 2016). Increased sensitivity for glucagon in brown adipose or white adipose tissue may also explain the heightened light‐cycle lipid oxidation rate we observed, as glucagon receptor activity appears to be dispensable for white‐adipose lipolysis (Vasileva et al., 2022), however, the enhanced metabolic rate of brown adipose or white adipose tissue (Darcy et al., 2020) in GH‐deficient mice may render physiological relevance to this pathway. Indeed, further study is necessary to determine if glucagon receptor action in adipose tissue is relevant in these animals.

It is important to consider that our analysis here was restricted to the female sex, and this constitutes a major limitation of this study. Sex has an important impact on longevity and interventions that manipulate longevity, as female GHRH‐KO mice are especially sensitive to the lifespan‐increasing effects of calorie restriction (Sun et al., 2013), and disruption of the GHR during late adolescence or adulthood significantly extends lifespan in females but not males (Duran‐Ortiz et al., 2021; Junnila et al., 2016). While this does handicap the generalization of these findings, our data suggest that, at least in females, this differential response to glucagon is physiologically relevant as we consistently observed differences in the response both upon exogenous glucagon administration and during periods of fasting. Future studies examining the contribution of glucagon signaling to longevity should include the male sex, to determine if sex differences exist in this signaling pathway. It is also important to note that we assessed our long‐term indirect calorimetry data in the context of total body weight, to include the effects of both lean mass and fat mass on whole body metabolism. Assessment of indirect calorimetry data in the context of lean mass, another commonly employed experimental approach, might allow for greater sensitivity in detecting changes in metabolic rate that may escape observation when total body weight is used. This strategy should be employed with caution in GH‐deficient models however, as the metabolic contributions of adipose tissue discussed above would be ignored. We also recognize that no quantification of gluconeogenesis or fatty acid metabolism was made in the present study, and that experiments directly measuring these processes (i.e., isotype tracer experiments) would strengthen our results. In the present study, we present in vivo assessments of these pathways in conscious, unrestrained, mice employing substrate tolerance tests and indirect calorimetry. While these experiments indirectly assess gluconeogenesis and fatty acid metabolism, they indicate that these pathways are differently regulated in GHRH‐KO mice under a physiologically relevant condition of minimal experimenter manipulation. Future studies should directly investigate these pathways to provide a more quantitative assessment of these differences.

In conclusion, we show that long‐lived female GHRH‐KO mice display a reduced glycemic response to exogenous glucagon, with reduced blood glucose elevations following glucagon administration. Consistently, we observed lower blood glucose elevations upon pyruvate or glycerol administration, reduced expression of gluconeogenic genes, and lower CREB phosphorylation during fasting. Despite this, the GHRH‐KO mice displayed a greater increase in energy expenditure in response to a glucagon challenge, and elevated lipid oxidation rates during fasting; however, a mechanistic explanation for this remains to be elucidated. We also observed an altered pancreatic islet composition, with comparable levels of α‐cells but reductions in β‐cells observed in the GHRH‐KO mice. Together, these findings suggest that in this long‐lived mouse model a differential response to glucagon, where there is less endogenous glucose production and increased fat utilization between feedings, at least partially mediates the unique responses to fasting in GH‐deficient and GH‐resistant long‐lived mice.

## AUTHOR CONTRIBUTIONS

Liou Y. Sun conceptualized the study, oversaw overall direction, and secured funding. A. Tate Lasher designed experiments, collected data, analyzed data, and took the lead in writing the manuscript. Both authors edited the manuscript and provided critical feedback that helped shape the research, analysis, and manuscript.

## FUNDING INFORMATION

This work was supported in part by the National Institute on Aging grants AG048264, AG057734, and AG050225 (L. Y. S.).

## CONFLICT OF INTEREST STATEMENT

All the contributing authors declared no conflicts of interest.

## Supporting information


Data S1:

## Data Availability

The data supporting the findings of this study are available from the corresponding author upon reasonable request.
