# Supplementary material for: Distinct physiological characteristics and altered glucagon signaling in GHRH knockout mice: Implications for longevity
Source: Aging Cell. 2023 Sep 4;22(12):e13985. doi: 10.1111/acel.13985 (PMC10726877; doi:10.1111/acel.13985)
Supplement: Supplementary file 1 — Data S1: [file ACEL-22-e13985-s001.docx]

**Supplemental Table 1** Antibodies used

| **Antibody** | **Dilution** | **Host** | **Vendor (cat #)** | **Application** |
| --- | --- | --- | --- | --- |
| Anti-glucagon | 1:3000 | Mouse | Sigma (G2654) | IF |
| Anti-insulin | 1:400 | Rat | R&D Systems (MAB1417) | IF |
| Anti-mouse AF488 | 1:500 | Donkey | Jackson Immunoresearch (715-545-150) | IF |
| Anti-rat Cy3 | 1:250 | Donkey | Jackson Immunoresearch (712-165-150) | IF |
| Anti-phospho-CREB Ser133 | 1:1000 | Rabbit | Cell Signaling Technology (9198) | WB |
| Anti-CREB | 1:1000 | Rabbit | Cell Signaling Technology (9197) | WB |
| Anti-GAPDH | 1:1000 | Rabbit | Cell Signaling Technology (5174) | WB |
| Anti-rabbit HRP | 1:2000 | Goat | Cell Signaling Technology (7074) | WB |

IF – Immunofluorescence

WB – western blot

AF488 – Alexa Flour 488 conjugated secondary antibody

Cy3 – Cy3 conjugated secondary antibody

HRP – horseradish peroxidase conjugated secondary antibody

**Supplemental Table 2** Primer sequences used

| **Gene** | **Forward 5’-3’** | **Reverse 5’-3’** |
| --- | --- | --- |
| GCGR | GTACGGCATCATAGCCAACT | CCAGCACTGAACATTCTCAAAC |
| G6PC | CGACTCGCTATC TCCAAGTGA | GGGCGTTGTCCAAACAGAAT |
| PCK1 | ATCATCTTTGGTGGCCGTAG | ATCTTGCCCTTGTGTTCT |
| PCX | CTGAAGTTCCAAACAGTTCGAGG | CGCACGAAACACTCGGATG |
| GLUT1 | GCAGTTCGGCTATAACACTGG | GCGGTGGTTCCATGTTTGATTG |
| GLUT2 | TCAGAAGACAAGATCACCGGA | GCTGGTGTGACTGTAAGTGGG |
| GLUT3 | ATGGGGACAACGAAGGTGAC | CAGGTGCATTGATGACTCCAG |
| GLUT4 | GCCCGGACCCTATACCCTAT | GGGTTCCCCATCGTCAGAG |
| ACACA | ATGGGCGGAATGGTCTCTTTC | TGGGGACCTTGTCTTCATCAT |
| ACACB | ACCAGCTTTATCCTGGGCTC | GACGGTGAAATCTCTGTGCAG |
| CPT1a | GACTCCGCTCGCTCATTCC | ACACCAGTGATGATGCCATTCT |
| CPT2 | CAGCACAGCATCGTACCCA | TCCCAATGCCGTTCTCAAAAT |
| PPARa | CGGTGTGTATGAAGCCATCT | TAAGGAACTCGCGTGTGATAAA |
| ACTB | CCAGTTGGTAACAATGCCATGT | GGCTGTATTCCCCTCCATCG |


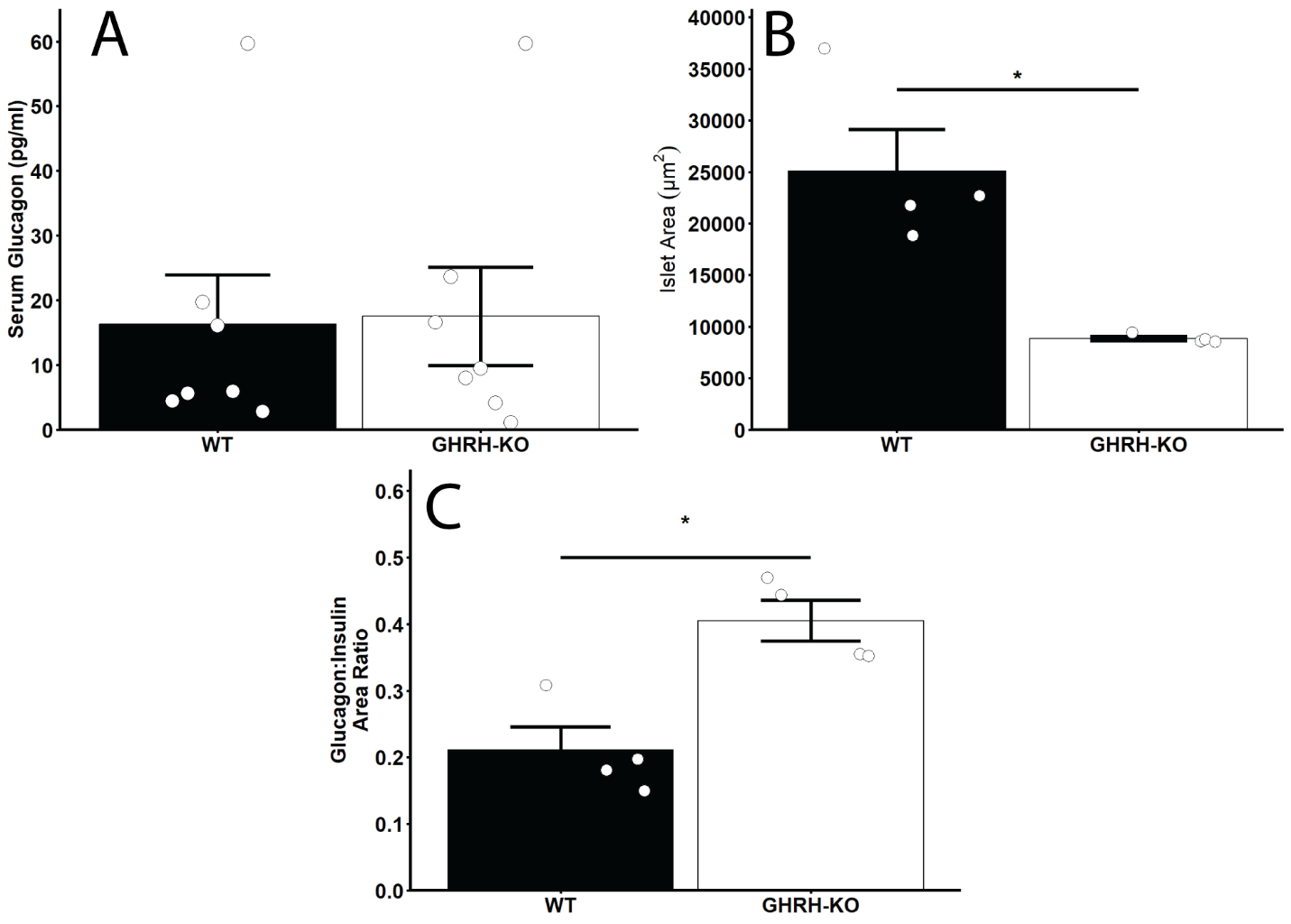


**Supplemental Figure 1.** Unchanged circulating glucagon and altered islet architecture in GHRH-KO females. Serum levels of glucagon in overnight (16 hours) fasted mice **(A)**, pancreatic islet area **(B)** and ratio of glucagon-staining area to insulin-staining area **(C)** measured during immunofluorescent microscopy in Fig. 2. Individual data points represent the average of all islets quantified for an individual. 6-10 islets were quantified per mouse per genotype. *p<0.05; ***p<0.001. Statistical significance was determined by two-tailed student’s t-test with the welch correction applied. N=7 mice per group **(A)** or N=4 mice per group **(B**, **C)**.


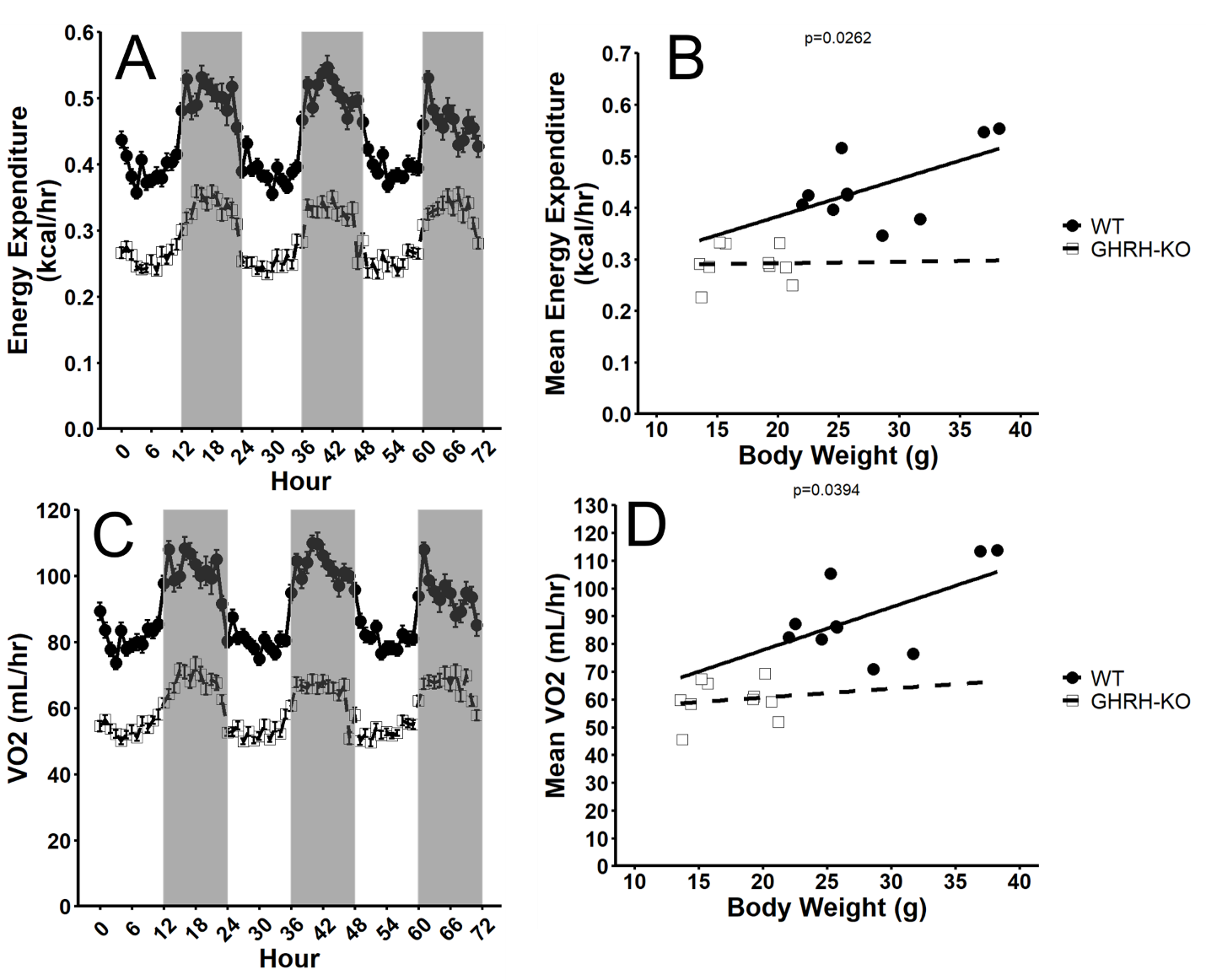


**Supplemental Figure 2.** Reduced absolute metabolic rate in GHRH-KO females. Unadjusted energy expenditure **(A)** and comparison of the influence of bodyweight on metabolic rate **(B)** for the duration of indirect calorimetry. Unadjusted VO2 **(C)** and comparison of the influence of bodyweight on VO2 **(D)**. Data presented as mean ± SEM or as data points representing individual mice**.** Data were analyzed by one-way ANCOVA, with bodyweight as a covariate and p-values representing the effect of genotype **(B, D)**. N=10 per group.


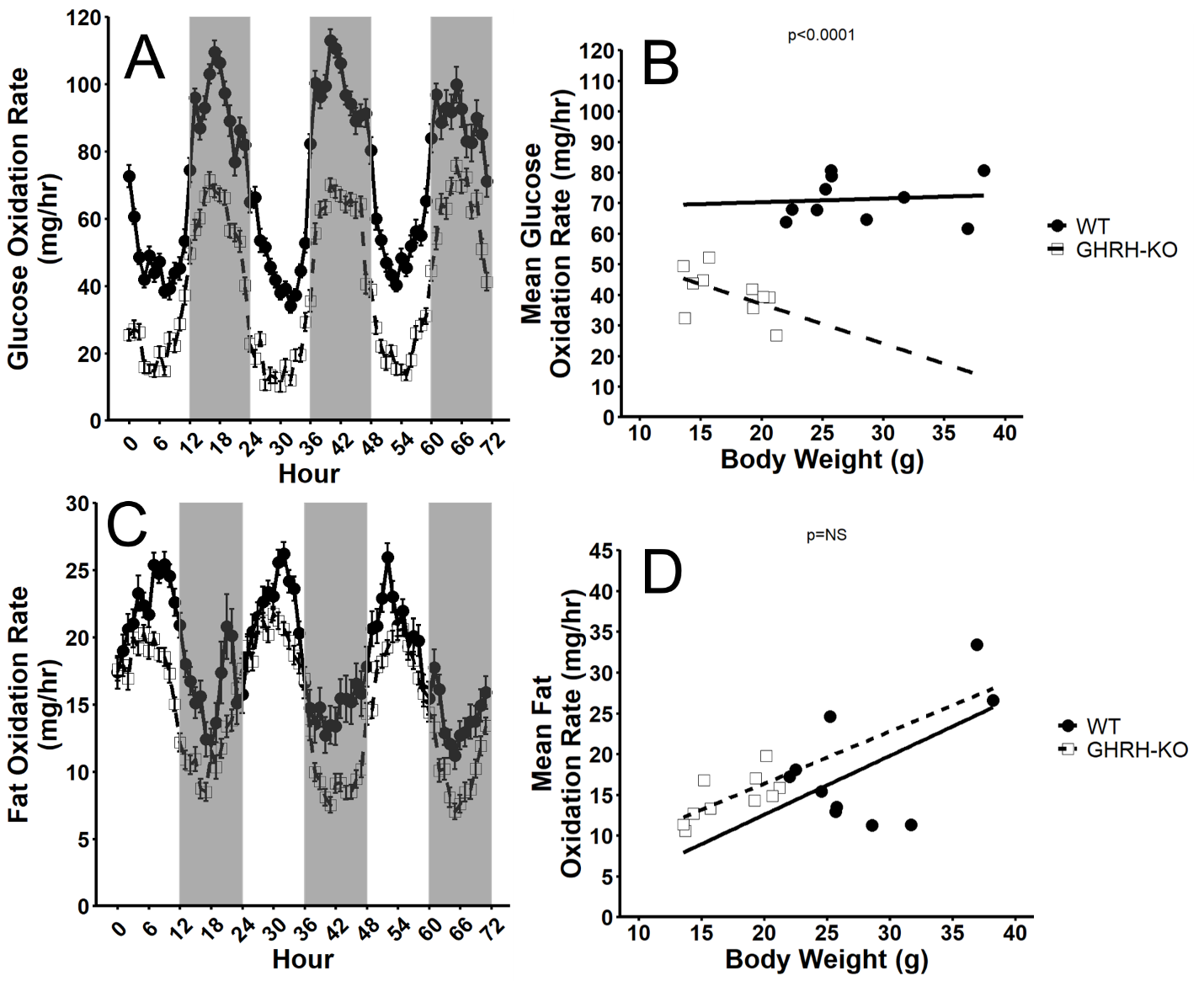


**Supplemental Figure 3.** Reduced absolute glucose oxidation rate, but comparable fat oxidation rate in GHRH-KO females. Unadjusted glucose oxidation rate **(A)** and comparison of the influence of bodyweight on glucose oxidation rate **(B)** for the duration of indirect calorimetry. Unadjusted fat oxidation rate **(C)** and comparison of the influence of bodyweight on fat oxidation rate **(D)**. Data presented as mean ± SEM or as data points representing individual mice**.** Data were analyzed by one-way ANCOVA, with bodyweight as a covariate and p-values representing the effect of genotype **(B, D)**. N=10 per group.


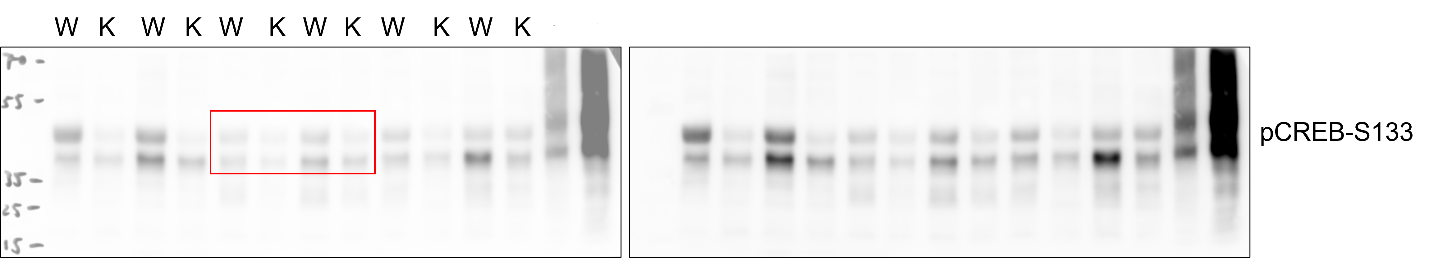


**Supplemental Figure 4.** Full membrane for phosphorylated CREB presented in Fig. 2J. Left panel represents light scanned blot with visible ladder and lane markings superimposed. Right panel is raw scan of image (no superimposition of ladders or lane markings). Final two lanes are CREB control cell extracts (CST #64854 and CST #73884) loaded according to manufacturer recommendations. W = Wild-Type; K = GHRH-KO.


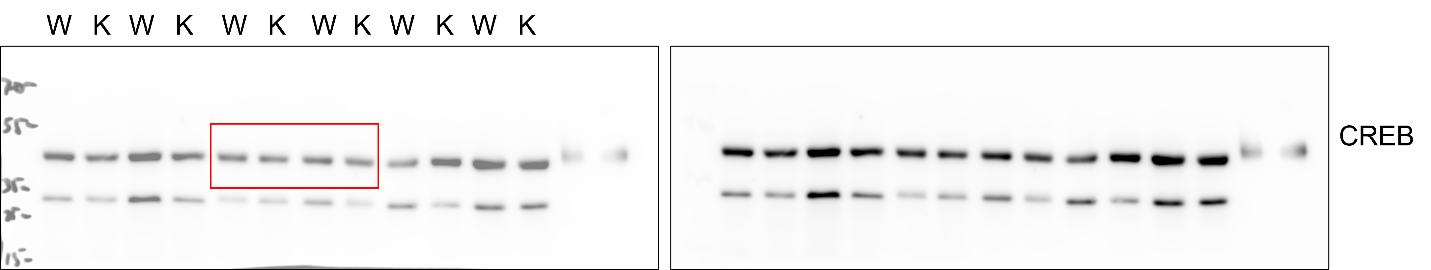


**Supplemental Figure 5.** Full membrane for total CREB presented in Fig. 2J. Left panel represents light scanned blot with visible ladder and lane markings superimposed. Right panel is raw scan of image (no superimposition of ladders or lane markings). Final two lanes are CREB control cell extracts (CST #64854 and CST #73884) loaded according to manufacturer recommendations. W = Wild-Type; K = GHRH-KO.


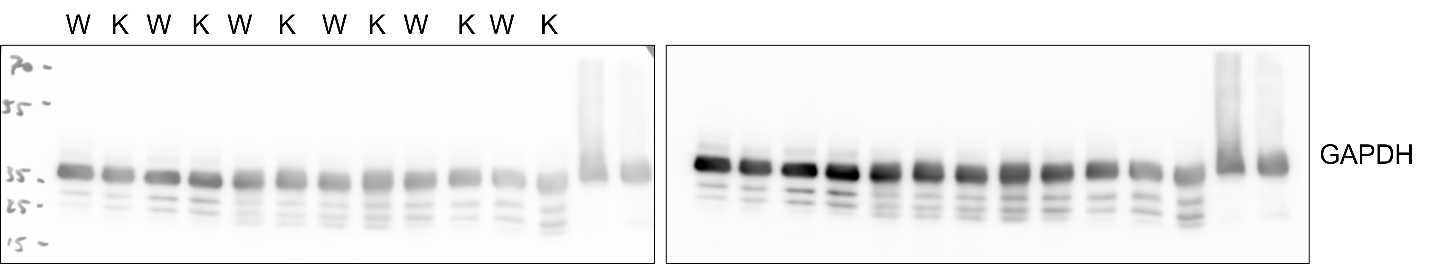


**Supplemental Figure 6.** Full membrane for GAPDH loading control presented in Fig. 2J. Left panel represents light scanned blot with visible ladder and lane markings superimposed. Right panel is raw scan of image (no superimposition of ladders or lane markings). Final two lanes are CREB control cell extracts (CST #64854 and CST #73884) loaded according to manufacturer recommendations. W = Wild-Type; K = GHRH-KO.
